# Relationship between breast tissue involution and breast cancer

**DOI:** 10.3389/fonc.2025.1420350

**Published:** 2025-04-07

**Authors:** Wenjing Li, Xian Zhao, Qinyu Han, Chuanxin Ren, Shang Gao, Yingying Liu, Xiangqi Li

**Affiliations:** ^1^ Department of Breast Center, The Second Affiliated Hospital of Shandong First Medical University, Tai’an, Shandong, China; ^2^ Department of The First Clinical Medical School, Shandong University of Traditional Chinese Medicine, Jinan, Shandong, China

**Keywords:** mammary gland, breast involution, TDLU, breast density, breast cancer

## Abstract

Breast tissue involution is a process in which the epithelial tissue of the mammary gland gradually disappears with age. The relationship between breast tissue involvement and breast cancer (BC) has received increasing amounts of attention in recent years. Many scholars believe that breast tissue involution is a significant risk factor for BC. Breast imaging parameters, particularly mammographic density (MD), may indirectly reflect the degree of breast tissue involution, which may provide a solid basis for classifying priority screening groups for BC. This review explored the relationship between breast tissue involution and BC by providing an overview of breast tissue involution and elaborating on the association between MD and BC. Consistent with the results of other studies, women with complete breast tissue involution had a lower risk of BC, whereas women with a high MD had a relatively greater risk of BC.

## 1 Introduction

Breast cancer (BC) is one of the most common malignancies in women and is the leading cause of morbidity, disability, and mortality in women worldwide (1). BC accounts for one-quarter of cancer cases and one-sixth of cancer deaths among women, leading the majority of countries in incidence and 110 countries in mortality (2, 3). Several risk factors are associated with BC development, including age, menopausal status, number of births, menopausal hormone therapy, and mammographic density (MD) (4); among these factors, the relationship between breast tissue involution and BC has garnered massive amounts of scholarly attention in recent years. Ginsburg et al. found that increased mammary gland density and delayed breast involution are associated with an elevated risk of breast cancer (5). Similarly, Radisky et al. demonstrated that stagnation of breast involution is a significant predictor of increased breast cancer risk (6). Herein, we review the relationship between breast tissue involution and BC.

## 2 Composition, development and involution of breast tissue

### 2.1 Basic composition of breast tissue

The mammary gland is a complex, branching tubular alveolar structure that is a major feature of mammals (7). The human breast has 15–20 lobes, each with many lobules containing alveoli, the secretory structures of the breast. The lobules are the basic building blocks of the mammary gland and include terminal ducts, alveoli, and the interstitium within the lobules; this is also known as the terminal duct lobular unit (TDLU) (8). TDLUs are traditionally evaluated qualitatively and classified into four lobule types: type 1 (least developed; <12 acini), type 2 (intermediate; ~50 acini), type 3 (fully developed; >80 acini), and type 4 (occurring during pregnancy and lactation). TDLU involution is a natural phenomenon that occurs with age as type 2 and 3 lobules regress to type 1 (9–11). TDLUs are the functional milk-producing structures of the mammary gland and the origin of most BC precursors and cancers (10). The lobules are surrounded by stroma containing adipocytes, endothelial cells, fibroblasts, and immune cells (12). These cells undergo extensive morphogenesis and regeneration throughout the mammalian life cycle.

### 2.2 Developmental process of breast tissue

The mammary gland is a highly dynamic organ that undergoes profound changes during puberty and during the reproductive cycle (13). The development of the mammary gland consists of four main stages: embryonic, pubertal, adult, and reproductive. The mammary gland is a highly dynamic organ, and from birth to puberty, it is in a relatively static state. At the onset of puberty, the original basal mammary epithelium expands rapidly to form an extensive ductal network through branching, elongation, and infiltration of the stroma (14). The mammary gland is under the influence of estrogen. It develops rapidly under the action of estrogen, and glandular lobule initiation gradually results in the formation of the TDLU. Progesterone increases the size of glandular lobules, and alveolar epithelial cells proliferate and undergo hypertrophic differentiation into cells with secretory functions. The adult mammary gland is stimulated by progesterone, and ductal complexity increases through the lateral branches in response to the cyclic estrous cycle (13). During pregnancy, ductal branches expand from the TDLU to form alveoli (15). During lactation, apical ductal epithelial cells synthesize and secrete milk proteins into the lumen of the alveolus, and oxytocin causes the surrounding myoepithelial cells to contract, transporting milk through the ductal tree to the nipple (16). When milk secretion ceases, whether due to the absence of breastfeeding after birth or following weaning, the mammary glands undergo atrophy (17). The elasticity of the female mammary gland begins to decrease at approximately 25 years of age, and the epidermis of the mammary gland begins to thin at approximately 40 years of age, eventually leading to changes in the morphology of the mammary gland (18). As women age, the breasts become more elastic and undergo a variety of structural and histological changes.

### 2.3 Involution of breast tissue

According to the concept of “breast tissue involution” proposed by Pike et al., the breast epithelium and mesenchyme should be affected by age, litter size, hormones, and other similar factors (19). As women age, they enter the perimenopausal period. Ovarian function then begins to decline from cyclic ovulation to intermittent ovulation or no ovulation at all. During perimenopause, changes in hormone levels in the female body are characterized by a decrease in progesterone and a relative increase in estrogen; glandular elements are gradually replaced by collagen and fat, resulting in fibrosis of the lobules and interstitial fibrosis within the lobules characterized by follicular epithelium (8). After menopause, in addition to the continued decline in progesterone levels, estrogen levels also begin to decrease. Both stromal and epithelial tissues are partially replaced by adipose tissue. The Pike model suggests that breast tissue degeneration occurs most rapidly at menarche, slows during pregnancy, decelerates further in the perimenopausal period, and is slowest after menopause (20). During lactation, the TDLUs develop into secretory structures, producing milk and forming lobuloalveolar structures, while surrounding adipocytes decrease in number. During the post-lactation recovery phase, breast tissue undergoes involution, with TDLUs returning to a pre-pregnancy state and no cumulative loss of glandular tissue (21, 22). Age-related lobular degeneration, or physiological atrophy of the mammary glands, is a process in which the number and size of each lobuloalveolar unit decrease, the interlobular stroma is replaced by dense collagen, and, ultimately, adipose tissue replaces the stroma (23).

**Figure 1:**
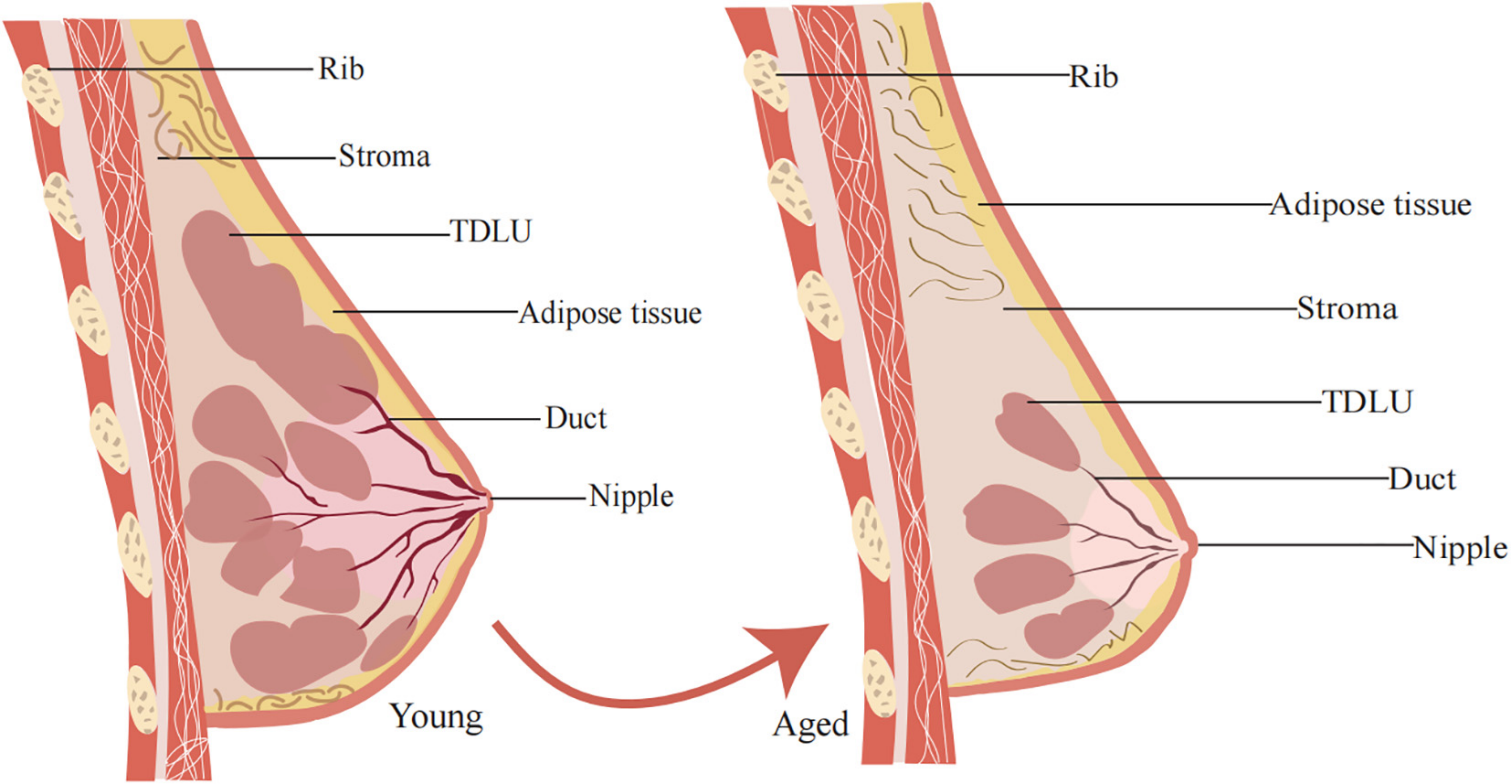
General structure of the mammary gland. The mammary gland consists mainly of outer epidermal tissue, inner fat and glandular tissue. Changes in mammary gland morphology during aging. With increasing age, the epidermis of the female breast continues to thin, the elasticity of the mammary gland decreases, and the mammary gland matrix becomes soft and undergoes ptosis as it is replaced by fatty tissue.

With aging, the number and size of TDLUs and lobules in the breast decrease, while adipose tissue significantly increases. This phenomenon is referred to as age-related TDLU involution (ARLI) (24). de Bel et al. found that breast tissue degeneration typically begins around the age of 30, with 45.6% of women under 30 exhibiting varying degrees of degeneration. In women aged 40 to 49, approximately 73.7% showed signs of degeneration (25). In most cases, nearly all lobular structures, which are the functional units (**Figure 1**) of the breast, are lost during this involution process. TDLUs are also the primary source of most breast cancer precursors and cancers (10, 22). During involution, type 2 and type 3 lobules degenerate into type 1 lobules. As a result, after menopause, type 1 lobules predominate, with type 3 lobules being relatively rare (9). Studies suggest that women with type 1 lobules, which are fully involuted, have a lower risk of breast cancer compared to women with type 2 or type 3 lobules (26).

## 3 Imaging of breast tissue degeneration

With advances in radiomics and deep learning, structural patterns of mammography can be quantitatively assessed and incorporated into risk models (27). Currently, mammography is a routine modality used in clinical practice for screening and diagnostic purposes (28). In addition, in high-risk women with a high MD, breast US and MRI have been demonstrated to detect occult cancers with mammographic exposure, providing independent risk indicators complementary to those of mammography (29).

### 3.1 Mammography

The appearance of the breast on a mammogram reflects the amount of fat, connective tissue, and epithelial tissue in the breast (30). Fat attenuates X-rays the least and appears black on mammograms, whereas mesenchymal and epithelial cells are dense tissues that attenuate X-rays more and appear white (5, 31). The concept of MD was introduced based on the different proportions of dense shadows in images (32). The decreases in collagen, glandular, and nuclear areas with age; lobular involution; and changes in MD are closely related phenomena, both of which are similar to Pike’s theoretical concept of “breast tissue aging” (5). Changes in MD are greater in younger, premenopausal women, while postmenopausal women have relatively lower MD (8, 33). MD grading based on the degree of MD is routinely used to characterize breast parenchyma, and a high MD is associated with a greater risk of BC (34). Accordingly, dense mammographic tissue is a strong risk factor for BC (35).

### 3.2 Breast ultrasound

Breast ultrasound can clearly reveal the milk ducts, fibrous connective tissue, and fat of the breast and can also detect earlier and smaller BC lesions in dense breast tissue (36). Sak et al. reported a significant correlation between MD and ultrasound findings (37). The glandular tissue component can be categorized as “low” or “high” based on the fact that it constitutes 50% of the fibroglandular tissue (FGT) in the breast. Glandular tissue composition was moderately positively correlated with MD: the higher the MD was, the greater the glandular tissue composition was. Studies have demonstrated that the composition of ultrasound-generated glandular tissue is negatively correlated with lobular involution of normal background tissue. Consequently, a high glandular tissue component reflects a large number of residual lobules and may represent the risk of fibroadenous tissue in BC (38). Hou et al. classified breast parenchyma into four types based on the different compositions of ducts, fibroglandular tissue, and fat lobules: heterogeneous type, ductal type, mixed type, and fibrous type (39). Ductal-type glands predominate in young women, mixed-type and fibrous-type glands predominate in middle-aged women, fibrous-type glands predominate in older women, and heterogeneous-type glands are observed in all age groups. Heterogeneous- and fibrous-type glands were found to be linked to BC risk. It has also been found that on mammography, the inner diameter of the milk ducts gradually decreases or even disappears with age, and the echoes gradually indicate a fibrous appearance (40). Consequently, it is possible to stratify the glandular tissue composition by ultrasound evaluation. The greater the glandular tissue composition on breast ultrasound was, the lower the degree of lobular regeneration and the greater the risk of BC.

### 3.3 Magnetic resonance imaging

MRI provides high-quality images of fat and FGT, the target tissues for MD measurement, based on three-dimensional imaging (41). The ratio of FGT to background parenchymal enhancement (BPE) in enhanced breast tissue correlates with BC risk. The FGT can be considered the MRI equivalent of the MD, reflecting the stromal and epithelial tissue components of breast tissue (42). On the other hand, BPE is defined as the enhancement of a normal breast FGT on breast MRI and is usually classified into the following four categories: minimal, mild, moderate, and severe, regardless of MD (43–45). Women with a high MD but minimal BPE do not have an increased risk of BC, suggesting that BPE may be a more accurate predictor of risk than MD alone (27, 46). King et al. also reported in their study that moderate or severe BPE was correlated with a greater likelihood of BC than minimal or mild BPE was (47–49). In addition, the findings of Dontchos et al. corroborate the above reports that women with moderate or significant BPE, as assessed by MRI, were nine times more likely to develop BC than women with mild BPE were (50). During normal aging, reproductive hormone levels decrease, a finding that is associated with involution in individuals with TDLU. Normal aging tends to decrease MD in women, and FGT is significantly lower. As BPE fluctuates in response to hormone levels, postmenopausal women have a significant decrease in both BPE and FGT. BPE decreases more significantly than FGT (51, 52). Conversely, abnormally high FGT or BPE in postmenopausal women indicates an increased risk of BC.

## 4 MD and breast cancer

The MD, the amount of radiopaque FGT relative to radiolucent adipose tissue on mammography, has been established as an imaging biomarker of BC risk and incorporated into a risk assessment model (38, 53). MD measurements are based on dense breast area and percent mammographic density (PMD), which is the percentage of dense breast area divided by total breast area (54). Wolfe described the relationship between the qualitative classification of MD and BC risk in 1976, and a large body of literature has now demonstrated that MD is linked to an increased risk of BC and is a distinct risk factor for BC (55, 56). Similarities exist between MD and Pike’s model of breast tissue aging (57). The model hypothesizes that the slowing of the rate of increase in age-specific BC incidence after menopause is ascribable to a decrease in the rate of breast tissue aging in postmenopausal women (58). In a recent analysis of MD data, researchers observed that MD decreased with age in premenopausal and postmenopausal women, and this decrease was more pronounced during menopause (20). Lokate et al. also reported that the percentage of MDs decreased with age (59). This difference may be attributed to the involution of the mammary gland, which is characterized by a continuous decrease in epithelial and stromal cells and an increase in adipose tissue (20, 60). Accordingly, less dense mammary glands degenerate more than denser mammary glands, and a higher MD is associated with increased stroma and decreased adiposity (61). Recently, there has been increased research on the relationship between MD and BC, and several studies have demonstrated that higher levels of MD are linked to a greater likelihood of breast and interval cancers (62). Most scientists also believe that dense breasts are a high risk factor for BC development, and this risk relationship is more pronounced in older postmenopausal women than in younger women (28).

PMD is also one of the strongest known risk factors for BC (63). Both dense areas and PMD were positively correlated with the risk of developing BC, with PMD being the stronger risk factor. A meta-analysis by Pettersson et al. revealed that dense areas on mammograms are associated with a reduced risk of BC (35). PMD decreases with increasing age, whereas breast cancer incidence increases with age (56, 64). As women age, the PMD decreases on average, while the incidence of BC increases; these findings are substantiated by the results of cross-sectional and longitudinal studies. To explain this apparent anomaly, Boyd et al. observed that in the model proposed by Pike to explain the age–incidence curve for BC, the decrease in PMD with age parallels the rate of senescence of breast tissue (54, 65, 66).

The 4th edition of the BI-RADS (**Table 1**) used percent density to assess risk: almost entirely fatty (<25% glandular); scattered (approximately 25–50% glandular); heterogeneously dense (approximately 51–75% glandular); and extremely dense (>75% glandular) density (67). A study by Boyd and McCormack et al. concluded that women with dense tissue in 75% or more of the breast have a risk of breast cancer four to six times greater than the risk among women with little or no dense tissue (57, 68, 69). Kavanaget et al. demonstrated that women with a high MD had a fivefold greater risk of cancer than women with a low MD did (70). According to the 5th edition of the BI-RADS (**Table 1**), the classifications are as follows: (a) almost entirely fatty; (b) scattered areas of fibroglandular density; (c) heterogeneously dense, which may obscure the detection of small masses; and (d) extremely dense, which lowers the sensitivity of mammography (71, 72). In general, women with grade C or D breasts are considered to have “dense breasts”. Researchers have shown that each 1% increase in MD increases the risk of BC by 3% and that women in the highest quintile of density change according to mammograms have a 3.6-fold increased risk of BC (73). In addition, Boyd and coworkers reported that women with a high MD had a 9.7-fold increased risk of atypical hyperplasia compared with women with a low MD (74). In contrast, women with a low MD, whose breast tissue was almost entirely fat, had a relatively low risk of future BC regardless of the histology of their breast biopsies (75). Accordingly, it appears that women with a high MD have a greater risk of developing BC. Additionally, the age-related decrease in MD may be due to mammary gland involution accompanied by a continued decrease in epithelial and stromal cells and an increase in adipose tissue. It can be inferred that women with less TDLU involution have higher levels of MD, i.e., a greater risk of BC.

**Table 1:** Categories of the American College of Radiology BI-RADS assessment of mammographic breast density.

BI-RADS, Edition and Category	4th edition	5th edition
Breast Tissue Characteristics	1 Almost entirely fatty (<25% glandular)	A The breasts are almost entirely fatty
	2 Scattered densities (approximately 25%–50% glandular)	B There are scattered areas of fibroglandular density
	3 Heterogeneously dense (approximately 51%–75% glandular)	C The breasts are heterogeneously dense, which may obscure small masses
	4 Extremely dense (>75% glandular)	D The breasts are extremely dense, which lowers the sensitivity of mammography

## 5 Breast tissue involution and breast cancer

In addition, studies from the Mayo BBD cohort have reported that an increase in the number of alveoli per lobule and the absence or partial involution of lobules are linked to a greater risk of BC (76, 77). The degree of TDLU involution with age has also been associated with various BC risk factors, including full-term pregnancy, breastfeeding (78), mammographic image density (79–81), and circulating hormone levels (82, 83), suggesting that TDLU involution may serve as a histologic marker of BC risk. Because TDLU is a major source of BC and its precursors, age-related decreases or delays in TDLU involution are associated with increased BC risk (78, 84). Delayed involution, i.e., a decrease in the rate or extent of reduction in the number and size of breast lobules with age, also contributes to BC development (8). Although postlactational involution usually has no pathological consequences, dysregulation of tissue architecture and activation of tumor microenvironmental features may also promote the growth of precancerous cells present in the breast (85).

### 5.1 Postpartum breast involution

The postpartum breast involution process occurs in two stages. The first stage is reversible and is characterized by the programmed cell death of alveolar cells, while the lobular-alveolar structure is preserved (86). After lactation ceases, transcription factor 3 (STAT3) is activated, and STAT3 regulates epithelial cell apoptosis by activating pro-apoptotic Bcl-2 family members, upregulating the PI3K inhibitory subunit, and downregulating MAPK survival signals (87). Additionally, STAT3 mediates the formation of triglyceride vacuoles, which induces lysosome-mediated programmed cell death, thereby promoting the involution process (88). Furthermore, the transforming growth factor (TGF-β3) produced during milk stasis can promote apoptosis. The second stage is irreversible, involving the degradation of the basement membrane and extracellular matrix (ECM) by proteases, breast remodeling, and the replacement of epithelial cells due to the differentiation and proliferation of adipocytes (89). Elder et al. discovered that SEMA7A may activate β1 integrin signaling during this stage, providing pro-survival mechanisms to overcome apoptosis (90).

Postpartum breast cancer (PPBC) refers to breast cancer diagnosed within a period following pregnancy and delivery, typically within one to ten years postpartum. It most commonly occurs within five years after childbirth, particularly in the first two years postpartum, when the incidence of breast cancer is notably higher (91). Research indicates that breast involution caused by weaning is a key driver of the increased incidence of breast cancer in young parous women. For instance, Lyons et al. found that postpartum breast involution promotes breast cancer progression via collagen and cyclooxygenase-2 (COX-2) (92). Guo et al. further showed that fibroblasts activated during involution (PDGFRα+ cells) promote PPBC and exhibit immunosuppressive activity (93). Macrophages are key immune cells in the postpartum breast remodeling process, and due to their role in tumor metastasis, they may contribute to the high metastatic potential of PPBC. Classical activated macrophages (M1 macrophages) typically inhibit tumor growth, while M2 macrophages promote tumor cell growth, invasion, and metastasis by secreting IL-10, TGF-β, and MMPs (94). During the peak of postpartum breast tissue remodeling, the number of M2 macrophages is six times higher than in non-lactating breasts, which may play a role in the initiation and progression of PPBC (95).

### 5.2 Age-related TDLU involution

**Figure 2:**
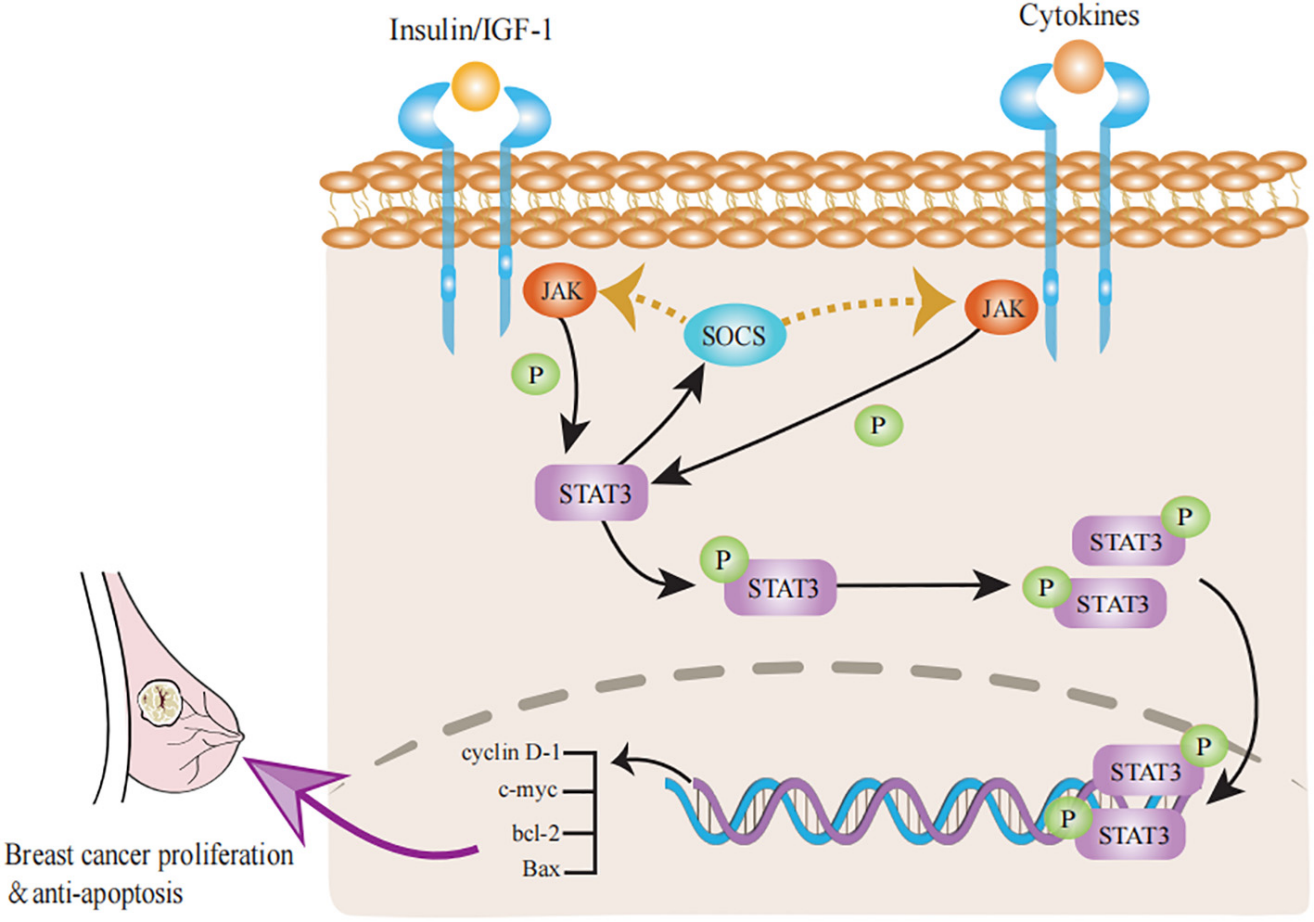
Progress of STAT3 signaling pathways in breast tissue involution. The classical JAK/STAT3 pathway can activate the transcription of cyclin D-1, c-myc, bcl-2 and Bax to promote proliferation and inhibit apoptosis in breast cancer. Activation of STAT3 signaling stimulates a negative feedback response through the induction of SOCS factors, which inhibit the activity of both insulin/IGF-1 receptors and many cytokine receptors. Accordingly, cytokine receptors can inhibit insulin/IGF-1 signaling through STAT3/SOCS signaling and induce insulin resistance.

With age, the mammary glands are affected by a number of changes in hormone levels that may be related to a decline in ovarian function. Normally, the levels of breast-related hormones and insulin-like growth factor (IGF)-1 decline with age, which may be linked to breast tissue involution (96). Zeina et al. examined the effects of hormones on TDLU involution in normal tissue and reported that in premenopausal women, high levels of oxytocin were correlated with high TDLU counts, whereas high levels of progesterone were associated with low TDLU counts. In contrast, in postmenopausal women, high levels of estradiol and testosterone were linked to high TDLU counts (97). Similarly, Khodr et al. reported that elevated estradiol levels were associated with increased TDLU counts after menopause, which is consistent with the growth-promoting role of estrogen in breast development and has been identified as a risk factor for BC in postmenopausal women (98). The findings of Fuhrman et al. are consistent with the above findings, with women with higher levels of circulating estrogen after menopause having a greater risk of BC (99). The potential mechanism for this phenomenon is that when estrogen levels are high, the risk of BC increases. The underlying mechanism is that estrogen binds to the ER to promote cell proliferation and reduce apoptosis, thereby maintaining high numbers of TDLUs in the breast and increasing BC risk (100). This confirms the characteristics of breast tissue involution, i.e., histologic loss of epithelial cells available for malignant transformation, which reduces the risk of BC (26, 77). In addition, growth hormone and insulin-like growth factor (IGF)-1 levels decline with age and are influenced by postmenopausal sex hormone changes (101). Rice et al. suggested that high levels of IGF-1 may inhibit breast tissue involution in women and increase the density of mammograms (102). IGF-1 signaling may control immunosuppression and cellular senescence through several links with STAT3 (**Figure 2**) signaling (103). The STAT3 signaling pathway induces cellular senescence through the STAT3/SOCS/p53 pathway (104, 105). STAT3 induces the expression of SOCS proteins, which inhibit the function of JAK/STAT3 signaling; for example, SOCS proteins exert negative feedback on JAK/STAT3 signaling induced by insulin/IGF-1 and certain cytokine pathways (106). As SOCS proteins inhibit insulin/IGF-1 signaling, they may induce insulin resistance, a condition known to be associated with aging. Inflammation stimulates the JAK/STAT3 pathway and increases the expression of SOCS proteins (107). IIS also plays a key role in promoting cell proliferation and inhibiting apoptosis (108). Reduced IIS is a common feature of physiological senescence and accelerated senescence (109). The IIS pathway not only plays a role in organismal senescence but also plays a regulatory role in TDLU involution. In particular, an increase in circulating IGF-1β is closely related to involution in TDLU cells and can induce breast disease during the aging process (110). Hormonal changes occur around menopause, particularly a decrease in estrogen and progesterone levels. Although the hormones associated with BC decrease with age, these hormones may be replaced by other hormones during aging, leading to an increased incidence of BC (111).

The proportion of mammary adipocytes increases with age, leading to increased aromatase secretion, mammary epithelial cell hyperplasia, and BC risk (112). With aging, the accumulation of adipose tissue in the mammary gland in close proximity to BC tissue may promote cancer cell growth and tumor metastasis by secreting factors and nutrients (113). Mammary macrophages play a pivotal role in the immune response, and their proportion decreases with age, leading to inactivation of the immune response and resulting in breast diseases such as mastitis, fibroadenoma, and BC (114). In addition, several studies have shown that highly expressed proinflammatory markers, such as TNF-α, COX-2, IL-6, CRP, leptin, SAA1, IL-8, and IL-10, are negatively correlated with the degree of lobular involution. Accordingly, high expression of breast inflammatory markers is associated with decreased lobular involution, which may increase the risk of BC (115). There have been conflicting findings regarding breast involution and BC risk. Milanese and colleagues classified involution extent as none (0% involuted lobules), mild (1–24%), moderate (25–74%), or complete (≥75%) and reported that increased lobular involution was associated with a decreased risk of breast cancer (77, 116). Figueroa and colleagues reported higher TDLU counts (i.e., less involution) in women with lower BMI, gestational age, or age at first birth (117). Kensler et al. reported similar results to those of Figueroa and colleagues (10). In summary, it was concluded that age at first birth, number of births, and birth interval birth indices were also negatively correlated with TDLU involution (118). Sherman et al. observed a positive correlation between estradiol and testosterone levels and TDLU counts in postmenopausal women (119). Pankratz et al. reported that exposure to factors such as smoking, which can reduce the effects of estrogen, was also associated with increased involution in patients with TDLU (120). Overall, incomplete or delayed involution of breast tissue has been linked to an increased incidence of BC and may be related to hormonal imbalances in the breast, such as abnormal levels of estradiol, testosterone, circulating IGF-1, and proinflammatory cytokines. However, the specific physiological mechanisms underlying the involution of the TDLU are not fully understood and require further study.

## 6 Discussion and conclusions

As women age, their breasts undergo a series of biological changes, including involution of TDLUs, an increase in MD and fat pads, hormonal changes, and cellular transformations. These changes are often associated with the development of breast-related diseases (60). TDLU involution is a physiological aging process in breast tissue characterized by a decrease in the epithelial component of the breast (121). It is characterized by a decrease in the ductal epithelium and is linked to the complexity and extent of the ductal epithelium (24). In the human mammary gland, involution of the TDLU with age results in a decrease in the TDLU size, total number of TDLUs, and number of alveoli per TDLU (110, 117). Age-related lobular involution differs from postlactational involution and is characterized by marked apoptosis and morphologic changes. Reduced age-related involution in the TDLU is negatively associated with BC risk (97). The mammary gland normally undergoes complete or near-complete physiologic atrophy, and the incidence of cancer steadily increases with age. However, these findings seem to contradict the conclusion that complete age-related involution of mammary epithelial cells reduces BC risk (77, 122). The continued increase in BC risk with age may be linked to the persistence of glandular epithelium beyond the normal time of involution, reflecting an abnormal delay in the aging process of the mammary gland (6, 122).

MD is a strong risk factor for breast cancer, and women with dense tissue occupying more than 60%-75% of the breast have a four- to sixfold-fold greater risk of breast cancer than women with little or no density (68, 123). The current study showed that most women without breast tissue involution have dense breasts. However, among women with complete lobular involution, the proportion of women in each category of MD was quite similar. One possible explanation is that in lobular involution, the atrophied mammary gland epithelium is replaced first by mesenchyme and later by adipose tissue. Consequently, complete involution with dense tissue on mammograms may indicate that despite epithelial atrophy, dense tissue reflects the contribution of stroma to MD (123). The increasing proportion of adipose tissue in the breast with age may be attributed to cytokines secreted by adipocytes that alter the tumor microenvironment (124). In addition, excess adipose tissue can form specific structures that can accelerate the conversion of androgens to estrogens, thereby triggering the potential development of BC (125).

In this review, we discuss the structure and development of breast tissue, imaging manifestations, density changes, and changes during involution. BC can originate from mammary epithelial cells, and an increase in the proportion of epithelial cells with age is correlated with BC incidence. Changes in the proportion of mammary cells and related gene expression during involution are strongly associated with an increased risk of BC. However, further studies are needed to investigate the relationship between involution-related breast diseases and changes in breast cells. In conclusion, breast tissue involution and MD are risk factors for BC; however, the relationship between these two conditions is still debated. Total or near-total atrophy of the organ, which is usually recognized as a factor associated with the increase in cancer incidence with age, occurs in more than 80% of women aged >50 years (126). However, the hypothesis that lobular involution of the mammary gland is a protective factor against BC seems to be contradictory, and the underlying mechanism is yet unclear. There is also a dearth of dynamic observational studies on the relationship between accelerated and delayed involution of breast tissue and BC, and whether differences exist between races requires further investigation. The current findings confirm that the normal involution of TDLU with age decreases the incidence of BC, whereas abnormal involution and increased MD lead to an increased incidence of BC; postpartum breast involution is also associated with an increased risk of breast cancer in young women. In addition, breast ultrasound, mammography, and breast MRI can indirectly reflect the degree of breast tissue involution, which may provide some basis for delineating the priority screening population for BC.
